# Influence of Nano-SiO_2_ on the Mechanical Properties of Recycled Aggregate Concrete with and without Polyvinyl Alcohol (PVA) Fiber

**DOI:** 10.3390/ma14061446

**Published:** 2021-03-16

**Authors:** Shenglin Wang, Baolong Zhu

**Affiliations:** Department of Civil Engineering and Architecture, Southwest University of Science and Technology, Mianyang 621000, China; wsltarget@163.com

**Keywords:** nano-SiO_2_, polyvinyl alcohol (PVA) fiber, SEM, recycled concrete, uniaxial compression, conventional triaxial compression

## Abstract

In recent years, recycled aggregate concrete (RAC) has become a research hotspot in the field of urban construction because of its resource utilization of construction waste. However, compared with original concrete, its strength is still low, which requires additional nano-SiO_2_ (NS) and fiber. In order to study the mechanism of strength improvement of RAC, this paper takes NS and polyvinyl alcohol (PVA) fiber as variable parameters; uniaxial and triaxial compression tests were carried out on RAC with PVA fiber and NS, and the mechanical properties of RAC were investigated The result shows that within the range of 3% NS content, an increase in the NS substitution rate causes the mechanical properties of RAC to improve significantly. The compressive strength of RAC increases again after adding PVA fiber; through a SEM (scanning electron microscopy) analysis of the specimen, it was found that the NS filled the micro-pores and micro-cracks in the RAC, and the PVA fiber changed the contact range between recycled aggregate and mortar, so the microstructure of the material was more compact. The mechanism of RAC strength improvement is explained in the microcosmic view.

## 1. Introduction

As urbanization gains increasing momentum, many buildings are demolished, resulting in substantial construction waste [1]. Meanwhile, construction of new buildings requires a lot of natural resources and consumes numerous natural aggregates. Encouraging the use of recycled concrete can help conserve natural resources and achieve the goals of sustainable development and environmental protection.

However, compared with natural concrete, recycled concrete has a much more complex structure. As recycled coarse aggregate boasts lots of micro-cracks, with the pressure of rolling, the old cement mortar and the aggregate will give rise to cracks. Influenced by the minor cracks in recycled aggregate, the interface transition coefficient of natural concrete is greater than recycled concrete [2]. In addition, the residual mortar left on recycled coarse aggregate boasts a comparably lower density and higher porosity, which lead to the recycled coarse aggregate exhibiting disadvantages such as lower strength and brittleness compared with natural concrete [3]. Meanwhile, as the polyvinyl alcohol (PVA) fiber is characterized with benefits such as environmental conservation, hydrophilicity, as well as corrosive and soluble base resistance, it possesses great adhesive strength with cement. At the same time, it serves as a concrete modification material, capable of improving the flexibility and impact resistance of concrete [4].

In recent years, surveys on recycled concrete mixing with PVA fiber have revealed that concrete with PVA fiber exhibits good ductility [5], inhibits the generation of micro-cracks and crack propagation, and narrows crack width [6]. At the same time, PVA fiber can minimize the stress concentration caused by the initial defects in recycled concrete materials, thus improving strength [7]. In terms of PVA fiber reinforcing concrete performance, varying amounts of fiber (0.05–0.38%) were added in the crack tensile tests of a standard PVA fiber concrete sample, which improved the plain concrete under the same circumstances [8]. Having thoroughly investigated the mechanical performance parameters of PVA fiber with regard to reinforcing concrete, Lin et al. found that the ideal PVA fiber content enhances the coherence of cement mortar and the compressive performance of the mortar [9]. Haskett et al. studied concrete mixed with PVA fiber and found that PVA fiber effectively prevents cracks from appearing in the concrete compression zone [10]. Yu et al. concluded that PVA fiber improves the compressive bearing capacity and fracture bridging capacity of concrete by improving the tensile toughness of the internal connection in concrete [11]. Having conducted tensile and other tests by adding PVA fibers into concrete, Cadoni et al. concluded that PVA fibers can significantly enhance the compressive strength and minimize the fracture and strain rate [12]. Additionally, Atahan et al. discovered that the flexural performance and impact resistance of composite materials alter significantly as the PVA quantity of fiber (at a mixing ratio between 0.5–2.0%) changes [13]. Meanwhile, the addition of PVA fiber also improves the impact toughness and durability of composite materials [14,15,16]. At present, most of the mechanical performance tests of PVA fiber on cementitious composite materials have confirmed increases in the porosity of the cement paste and the flexural strength of concrete [17,18]. 

Additionally, abundant studies have shown the capacity of nano-SiO_2_ (NS) in enhancing the mechanical properties and durability of concrete. The addition of NS into the concrete accelerates the hydration of cement, which reduces the consumption of cement, and improves the concrete strength from the micro perspective [19]. Generally, as the addition of NS is less than 3%, the compressive strength and flexural strength of concrete within 28 days can be increased by 10% and 25%, respectively [20]. 

Gonzalez observed that with a mixture of 2% NS, the compressive strength of the cement mortar and the bending stiffness were improved by 50% and 16%, respectively [21]. Gesoglu et al. [22] pointed out that the addition of 2% NS improves the tensile strength of concrete by 37.5%. Givi et al. found that the rise of NS enhances the flexural, compressive and tensile strength of the concrete [23]. By examining the effect of NS on the mechanical properties and porosity of concrete, Zhang et al. found that as the content of NS rises from 0.5 to 5%, the cracks of the samples gradually expand, and the internal NS generated by the unhydrated cement fills the pores [24]. Given the disadvantages of recycled coarse aggregate, such as low density, high porosity, low strength and brittle failure, it is of great significance to investigate the influence of PVA fiber and NS on the mechanical properties of recycled aggregate concrete. Previously, Mahmoud et al. proved that NS can improve the performance of martial fiber concrete and increase the compressive strength [25]. Sikora et al. studied the changes in the mechanical properties of acrylic polymer concrete following the addition of NS [26]. Ling et al. considered the influence of PVA fiber content and NS on the mechanical properties of recycled aggregate concrete (RAC). The results show that PVA fiber can enhance the flexural strength of concrete, and the addition of NS mainly affects the workability and tensile strength [27]. Mukharjee et al. have carried out experimental research on the replacement of Portland cement with nano colloidal silica. The results show that the compressive and tensile strength of the material are improved. When a small amount of NS is added, the performance of RAC is similar to that of original concrete [28]. However, few studies exist concerning the mechanical properties of PVA fiber and NS reinforcing recycled aggregate concrete. 

Based on previous studies on recycled concrete, PVA fiber concrete and NS concrete, this paper makes the uniaxial and triaxial compression tests on NS recycled aggregate concrete with and without PVA fiber and studies its impact on the work performance, mechanical strength and ultimate stress of the concrete from a macro perspective. Utilizing scanning electron microscopy (SEM), we discuss the influence of PVA fiber and nano NS on the microstructure of recycled aggregate concrete, which shows that the microstructure performance is in good agreement with the macroscopic mechanical properties. This paper also illustrates how NS fills the micro-pores and micro-cracks of the recycled aggregate, and how PVA fiber improves the contact surface of the new and old mortar. To sum up, the collaboration of PVA fiber and NS enhances the strength of recycled concrete, providing an efficient and effective purpose for its application. 

## 2. Materials and Methods

### 2.1. Test Raw Materials

The sample fibers used in our work are PVA fibers (10–20 mms in length, Jiangsu Capability Technology Co., Ltd., Nanjing, China), and Table 1 shows the physical and mechanical properties of the sample fibers. The cement is PO42.5R common Portland cement. The recycled aggregate comes from abandoned construction concrete that has been artificially broken, with an original strength of C30. The concrete was crushed by the jaw crusher into 3–10 mm particles and then sieved. With a sand ratio of 50%, the fine aggregate consists of particles with an average diameter of 0.44 mm and a mud content below 2.5%. NS is the solid white powder with particles 5 nm in diameter. This is dedicated for VK-SH30 concrete, with a purity of 99.5%, a specific surface area within 150 to 200 m^2^/g and a pH within 5 to 7. 

### 2.2. Experiment Mixture Proportions

**Figure 1:**
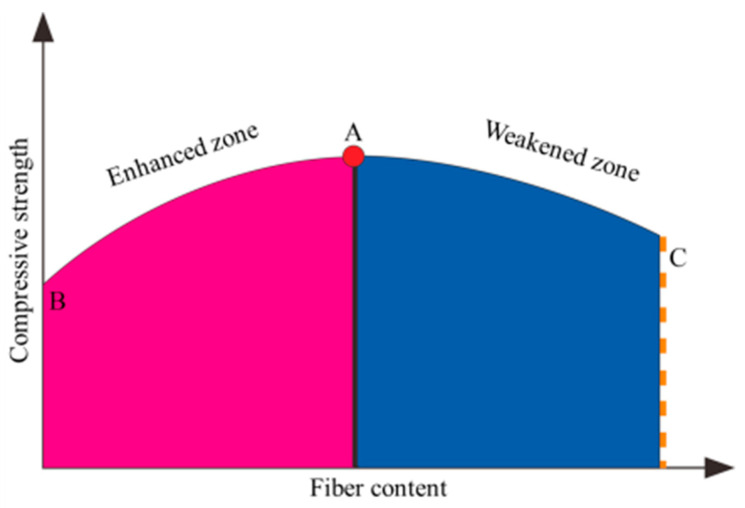
PVA fiber improves concrete strength [8].

The introduction has described the current research towards PVA fiber concrete and summarized that PVA fiber enhances concrete strength [8,9], as shown in Figure 1. With the design parameter of the concrete remaining unchanged, the rise in the quantity of PVA fibers enhances the strength of the sample from Point B to Point A, and thus the BA segment is regarded as the rising phase. As the fiber content surpasses Point A, the concrete strength gradually decreases to C due to internal reasons. Therefore, the AC segment was called the descending phase. As a result, the fiber content in our test was set at 3.6 kg/m^3^.

The design of concrete mix ratio is shown in Table 2. In which the NS content is 0%, 0.5%, 1.0%, 2.0%, 3.0% and 4.0% of the cement quality, respectively, RCN is recycled aggregate concrete mixed with NS, RCN-P is RCN mixed with PVA fiber, and the number after the code N represents the percentage of NS content.

### 2.3. Test Methods

**Figure 2:**
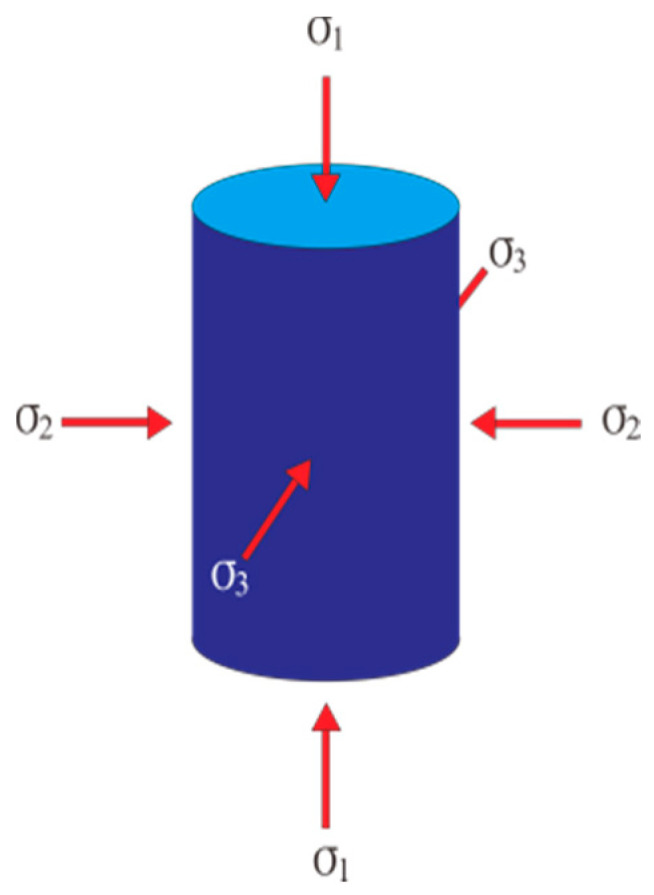
Stress test model.

With the completion of curing, all samples went through uniaxial compression and traditional triaxial compression tests. Figure 2 illustrates the stress test model.

After casting formation, the test piece was put into a standard curing room ((20 ± 2) °C, (95 ± 3)% RH) for 3 days, and then the mold was removed. As the test block without mold continued to stay in the standard curing room for a certain period of time, the test samples were examined on the DYS-2500 high temperature and pressure rock triaxial testing machine (Sichuan Dexiang Kechuang Instrument Co., Ltd., Chengdu, China), with 3 samples subjected to 3 values of confining pressures (0, 5, 10 MPa). Finally, the average of the test results was regarded as the final value. The loading path involves 2 stages: First, the confining pressure continued to increase at a speed of 0.25 MPa/s until the target confining pressure was reached; Second, under a constant confining pressure, the displacement rate was controlled at 0.06 mm/s.

## 3. Experiment Results

### The Failure Form of the Sample

**Figure 3:**
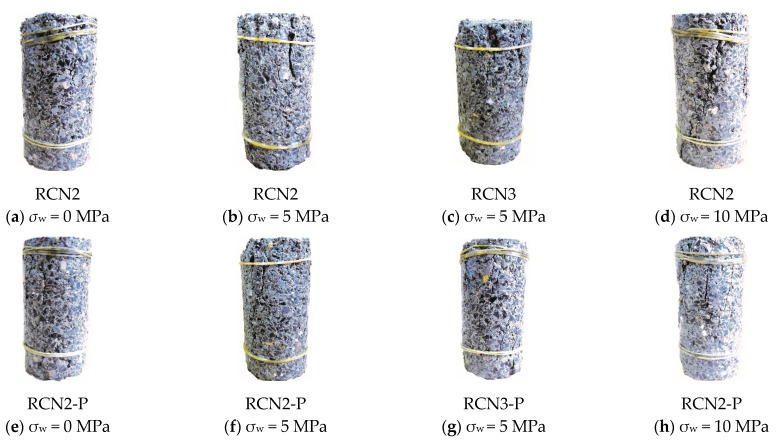
The failure mode of recycled concrete after compression.

By comparing the failure modes of NS-recycled aggregate concrete with PVA fibers and the concrete without PVA fibers under the confining pressure, we found that the failure mode of the sample was related to the content of NS, the value of the confining pressure and whether it contained PVA fiber. Figure 3 shows the partial failure of the sample.

Among them, the sample exhibited vertical splitting failure under uniaxial compression and oblique shear failure under triaxial compression. As there was no confining pressure in the lateral direction, the concrete was under uniaxial compression. With the content of NS within 3%, an increased content of NS gradually increased the elastic limit value of the recycled concrete and damaged the aggregate concrete. Furthermore, the addition of PVA fiber further improved the elastic limit value of the recycled concrete. As the load exceeded the elastic limit value, vertical cracks appeared in the middle of the test piece and extended to both ends and, at the same time, new small transverse cracks were generated. Compared with NS-recycled aggregate, the one mixed with PVA fiber produced fewer small cracks, and when the load reached the maximum, the bearing capacity of the sample lasted longer, but all the final samples showed split failure.

In triaxial compression, as the sample withstood the confining pressure from the lateral restraint, the interior structure could endure greater pressure. In contrast with uniaxial compression, the sample showed larger and more cracks. With the increase of the NS, the sample exhibited large cracks, but the width of the small cracks decreased. In the face of the corresponding confining pressure and NS replacement ratio, the PVA fiber could shoulder the load with concrete as the PVA fibers were arbitrarily allocated in the sample to construct a web system. Compared with the NS-recycled aggregate concrete, the concrete blended with PVA fiber showed higher durability and more compact cracks. Observation of the failure surface revealed that when the confining pressure value was 5 MPa, the interface between the recycled coarse aggregate and the cement paste was mainly sheared, but the coarse aggregate was hardly sheared. As the confining pressure value rose to 10 MPa, the recycled aggregate and the cement collision between the recycled aggregates were sheared and destroyed.

In conclusion, the failure mode of the specimen changes significantly with the increase of the confining pressure value. When the confining pressure value is 0, it is mainly the splitting failure of the interface between the coarse aggregate and cementitious body; with the increase of the confining pressure value, the vertical cracks gradually change into oblique cracks, and when the confining pressure value reaches 10 MPa, shear failure appears on the recycled aggregate. Compared with the failure mode of RAC without PVA, the aggregate at the failure surface of RAC with PVA is almost sheared into powder; at the failure surface of RAC with low nano silica content, the cement mortar wrapped with coarse aggregate is crushed into fine powder, and a small amount of coarse aggregate is sheared off.

## 4. Discussion

### 4.1. Analysis of the Compressive Strength under Uniaxial Compression

**Figure 4:**
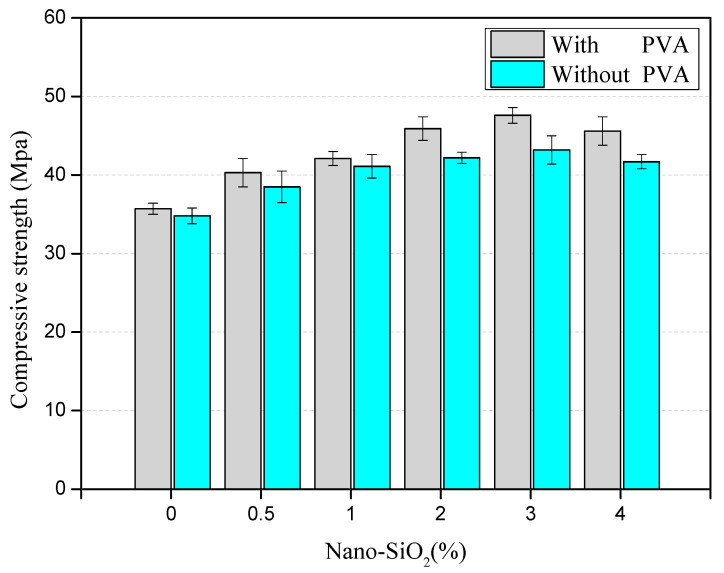
Compressive strength of nano-SiO_2_ (NS)-recycled aggregate concrete with and without PVA fibers.

The compressive strength of the NS-recycled aggregate concrete with and without PVA fibers was compared, and the results are shown in Figure 4.

Figure 4 shows the uniaxial compressive strength of the concrete improves as the NS content increases within is within 3%. When the content of NS is 3%, the strength of the recycled aggregate concrete is 15.1% higher than that without NS, which indicates that NS can improve the compressive strength of the concrete.

When the content of NS is 3%, a 7.6% increase of the compressive strength is registered by the NS-recycled aggregate concrete containing PVA fibers. It shows that the addition of PVA fibers enhances the compressive strength of NS-recycled aggregate concrete. When the NS content remains low, PVA fibers exert minor impacts on the NS-recycled aggregate concrete. Meanwhile, with the rise of NS content, the compressive strength of NS-recycled aggregate concrete blended with PVA fiber is gradually improved, and NS is easily dispersed in the course of mixing the concrete. Thus, NS is prone to penetrating the interface of recycled aggregate, with the effective release of NS.

Figure 4 also shows that when the NS content climbs up to 4%, the compressive strength of NS-recycled aggregate concrete begins to decrease, which illustrates that 3% NS content is capable of repleting the micro-pores and cracks of the recycled aggregate. As the NS content exceeds 3%, excessive NS content brings more pores in the matrix, thereby reducing the compressive strength.

### 4.2. Conventional Triaxial Test Analysis

#### 4.2.1. Stress-Strain Relationship Curve

**Figure 5:**
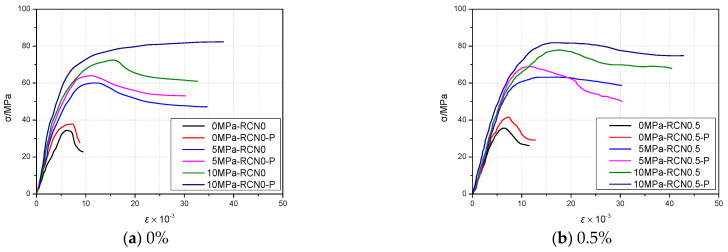

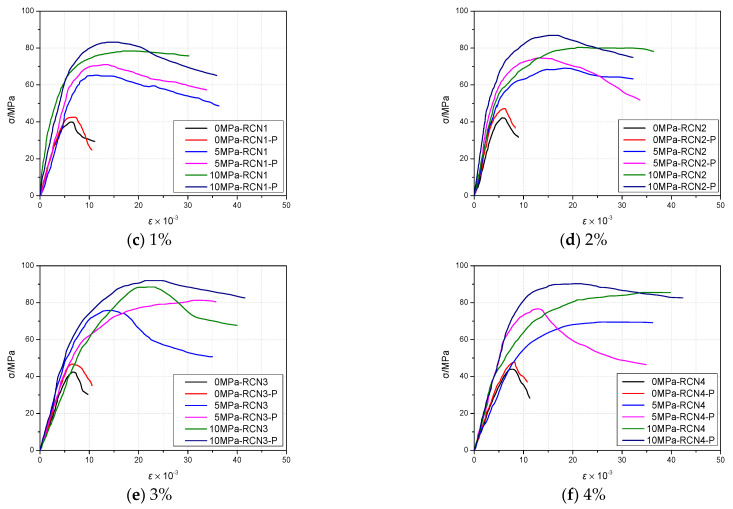
Changes of NS content under different confining pressures and stress-strain relationship curves of recycled aggregate concrete with and without PVA fibers.

Figure 5 presents the stress-strain relationship curve of the sample under different confining pressures. According to the data, as the lateral confining pressure value grows greater, the damage occurs later, and the strain during damage failure grows larger, which indicates that the lateral confining pressure value can virtually inhibit the occurrence and development of internal damage in recycled concrete. Under the confining pressure, no obvious difference is observed in the occurrence of the initial strain between the NS-recycled aggregate concrete with PVA fiber and the one without. Under triaxial compression, at the initial stage of loading, all samples are in the linear elastic stage under triaxial confining pressure. As they continue to load, the lateral confining pressure causes differences in the stress-strain process of the concrete samples, with the following main characteristics:

(1) As the confining pressure exists, the stress-strain curve turns to develop smoothly without an obvious peak, which shows that the deformation of the sample and the micro-cracks in the section under the confining pressure can be virtually restrained. As the confining pressure rises from 0 MPa to 10 MPa, the ultimate strain range of the sample will be regulated. After the ultimate stress interval, the stress-strain curve presents plastic features; however, as the applied load surpasses the appropriate axial stress and reaches the utmost strength of the concrete, the sample is instantly squashed. In other words, the stress-strain curve drops abruptly.

(2) With the confining pressure unchanged and the increase of the NS replacement ratio, the main compressive stress of the sample increases, along with a corresponding decrease in the axial strain. All in all, the curves of different NS replacement ratios show the same trend. When the NS replacement ratio remains constant, the rise of confining pressure leads to an increase in the main compressive stress and the axial strain. In contrast with the NS recycled aggregate concrete blended without PVA fiber, the concrete without fibers has a larger main compressive stress but a smaller axial strain. Owing to the confining pressure, the sample shows stronger plastic capacity and lasts longer in the triaxial test before it fails.

#### 4.2.2. The Influence of Confining Pressure on Peak Stress

**Figure 6:**
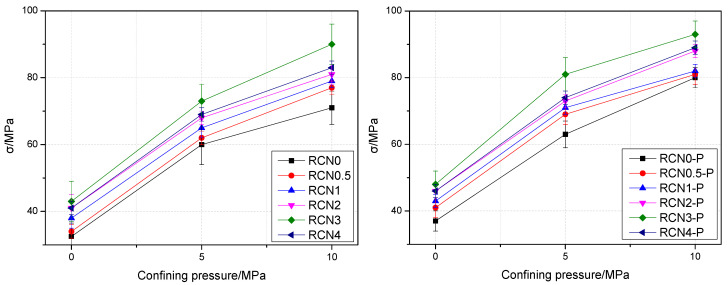
Relationship curve between confining pressure and peak stress.

Figure 6 shows that the lateral confining pressure has a large impact on the peak stress of concrete. The peak stress increases in varying degrees with the rise of the confining pressure. With the NS content controlled at 3%, when the confining pressure value rises from 0 to 10 MPa, the peak stress of the NS-recycled aggregate concrete containing PVA fibers soars by 211.4%, and the one without fibers by 193.7%. Therefore, the restraining effect of confining pressure greatly improves the bearing capacity of the concrete.

Comparing the peak stress of NS-recycled aggregate concrete with and without PVA fiber, it is found that under the same loading conditions, the strength of the former is higher than the latter, which is attributed to the addition of PVA fibers. As micro-cracks are generated in the aggregate while crushing, the strength of the aggregate is reduced, but the PVA fibers restrain the development of internal cracks and weaken the impact of micro-cracks in the aggregate.

Figure 6 demonstrates that when the confining pressure remains constant, the peak stress of recycled aggregate concrete continues to change with the rise of the NS replacement ratio. As the NS content gradually reaches 3%, the overall peak stress witnesses an increase. As the NS replacement ratio remains unchanged, the concrete peak stress of the RCN group and RCN-P group shows a linear trend with the rise of the confining pressure. Under the condition of NS substitution, with the rate of substitution and confining pressure unchanged, the peak stress of the NS-recycled aggregate concrete with PVA fibers is 1.21–1.40 times that of the samples without PVA fibers.

#### 4.2.3. SEM Microstructure Analysis

**Figure 7:**
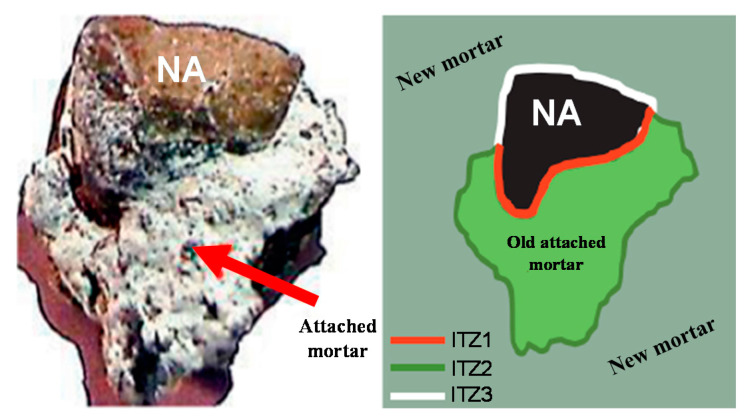
Schematic of interface transition zones [29].

Figure 7 divides recycled aggregate concrete into three transition zones [29]: (1) the transition zone between the recycled aggregate and the old mortar stuck to the recycled aggregate; (2) the transition zone between the new and old mortar; (3) the transition zone between the recycled aggregate and new mortar.

**Figure 8:**
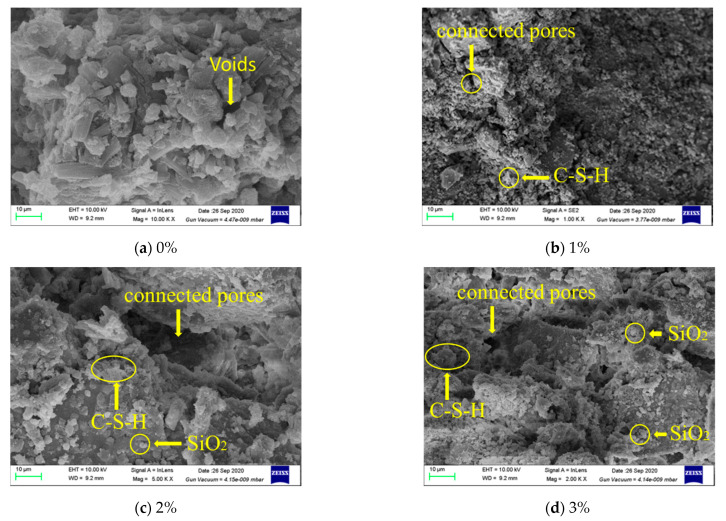
SEM images of the microstructure of recycled concrete with different NS contents.

Figure 8 shows the SEM images of the microstructure of recycled aggregate concrete with different NS contents (0%, 1.0%, 2.0%, 3.0%). Figure 8a presents the microstructure without NS, and it can be seen that the strengthened result has formed a network structure of strengthened calcium silicate (C-S-H). Meanwhile, Figure 8b–d demonstrate that the process of cement hydration is promoted to varying degrees, and the density of cement stone continues to rise with increased NS content. In this way, NS promotes the formation of a C-S-H gel, which is consistent with the conclusion obtained from the test results of Roncem [30]. Therefore, NS improves the strength of cement, cement mortar and concrete.

**Figure 9:**
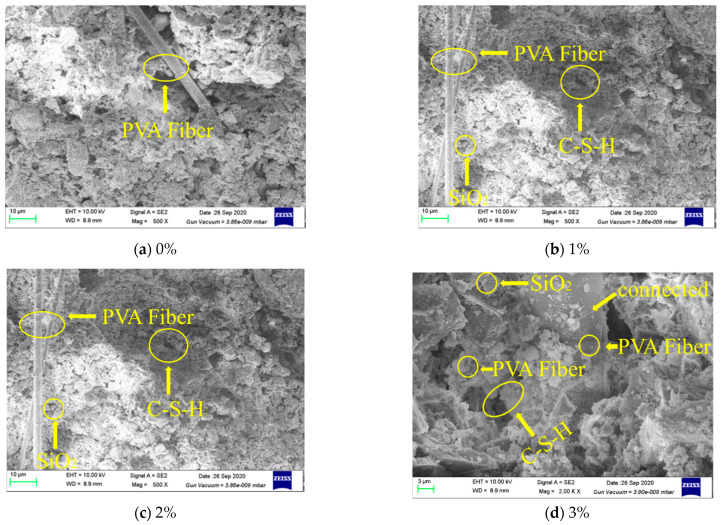
SEM images of the microstructure of recycled concrete with different NS contents and PVA fibers.

Figure 9a,b are the SEM scanning results of the microstructure of NS-recycled concrete mixing with PVA fibers. With the incorporation of PVA fibers into the cementitious material, the microstructure of the material appears as a network structure. As the NS content increases, the network structure of reinforced C–S–H and PVA fibers tend to grow denser. When PVA fibers are incorporated, the PVA fibers and NS will work together, resulting in obvious ettringite (AFt) crystals. NS particles and PVA fibers can be found in the sample matrix, and significant amounts of reinforced C-S-H is found in the interface transition zone, which indicates that NS particles and PVA fibers can be distributed in the interface transition zone of recycled aggregate concrete. Obvious micro-cracks are observed in the transition zone of the concrete interface with a width of several nanometers, which may be related to the NS entering the recycled aggregate. Figure 9c,d illustrate that the mix of NS with recycled aggregate concrete has greatly improved the compactness of the interface transition zone, and few large micro-cracks are present in the interface transition zone. This proves that the incorporation of NS and PVA fibers can significantly enhance the interface transition zone of recycled aggregate concrete. To a certain extent, this phenomenon explains how the addition of NS and PVA fibers enhance the compressive strength of recycled aggregate concrete.

**Figure 10:**
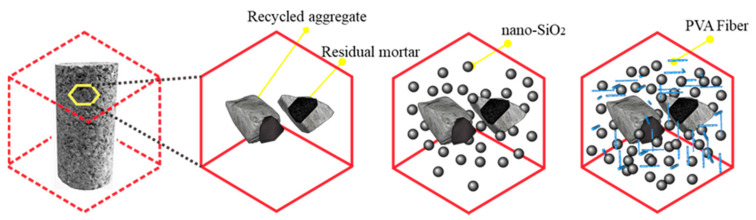
Synergistic mechanism of PVA fiber and NS.

Figure 10 shows the collaboration mechanism of PVA fiber and NS. As PVA fibers are incorporated into the recycled aggregate, the collaboration of PVA fiber and NS will increase the adhesive strength of the new and old mortar as well as the aggregate in recycled concrete, thus increasing the compressive strength of recycled concrete.

## 5. Conclusions

By changing the replacement ratio of mixed NS (0%, 0.5%, 1%, 2%, 3%, 4%) macroscopically, we compared the NS recycled aggregate concrete with PVA fibers and the one without in terms of mechanical properties, the failure process, deformation and other characteristics; meanwhile, the microstructure changes were analyzed by the SEM technique microscopically. In this way, we reached the following conclusions:

(1) The addition of NS within a certain range greatly improves the mechanical properties of the concrete. However, as the NS content exceeds a threshold, the performance of concrete no longer improves for the dispersion of nanoparticles.

(2) The synergistic effect of PVA fibers and NS improves the recycled aggregate concrete, which enhances the adhesive strength between new and old mortar, as well as aggregate in recycled concrete. Thus, the recycled concrete containing PVA fibers and NS shows higher compressive strength than that mixed with NS alone.

(3) When the NS content is 3.0%, the compressive strength of concrete without and with PVA fiber reaches 43.2 MPa and 47.6 MPa, respectively. The addition of PVA fibers improves the compressive strength of recycled aggregate concrete containing different contents of NS. As the NS replacement ratio remains constant and the confining pressure rises, NS-recycled aggregate concrete with and without PVA fibers will show a larger principal compressive stress but their axial strain is smaller. Comparison of uniaxial compression test and triaxial compression test, the triaxial compression test shows that NS-recycled aggre-gate concrete with PVA fiber improves its containing elastic characteristic and the specimen will take longer to fail.

(4) As the replacement ratio of NS remains constant and confining pressure increases, the peak stress of the concrete in the RCN group and the RCN-P group shows a linear increase. Under the condition of NS substitution, with rate substitution and confining pressure unchanged, the peak stress of the NS recycled aggregate concrete with PVA fibers is 1.21–1.40 times that of the concrete without PVA fibers.

## Figures and Tables

**Table 1:** Basic parameters of polyvinyl alcohol (PVA) fibers used in this study.

Young’s Modulus	Elongation at Break	Young’s Modulus	Fiber Diameter	Density	Tensile Strength
(GPa)	(%)	(GPa)	(μm)	(g/cm^3^)	(MPa)
36	6	36	21	1.30	1280

**Table 2:** Proportion of sample.

Numbering	Concrete Mix Design /(kg/m^3^)					
	Nano -SiO_2_ (kg/m^3^)	PVA Fiber (kg/m^3^)	Recycled Aggregate (kg/m^3^)	Portland Cement (kg/m^3^)	Water (kg/m^3^)	Silica Sand (kg/m^3^)
RCN0	0	0	1400	340	180	600
RCN0.5	1.5	0	1400	340	180	600
RCN1	3.	0	1400	340	180	600
RCN2	6	0	1400	340	180	600
RCN3	10	0	1400	340	180	600
RCN4	12	0	1400	340	180	600
RCN0-P	0	3.6	1400	340	180	600
RCN0.5-P	1.5	3.6	1400	340	180	600
RCN1-P	3.	3.6	1400	340	180	600
RCN2-P	6	3.6	1400	340	180	600
RCN3-P	10	3.6	1400	340	180	600
RCN4-P	12	3.6	1400	340	180	600

## Data Availability

Data sharing is not applicable to this article.
